# Supplementary material for: Amorphous silicon resistors enable smaller pixels in photovoltaic retinal prosthesis
Source: bioRxiv. 2025 May 7:2025.05.01.651774. Preprint. [Version 1] doi: 10.1101/2025.05.01.651774 (PMC12248045; doi:10.1101/2025.05.01.651774)
Supplement: Supplement 1 [file media-1.pdf]

Supplementary Materials

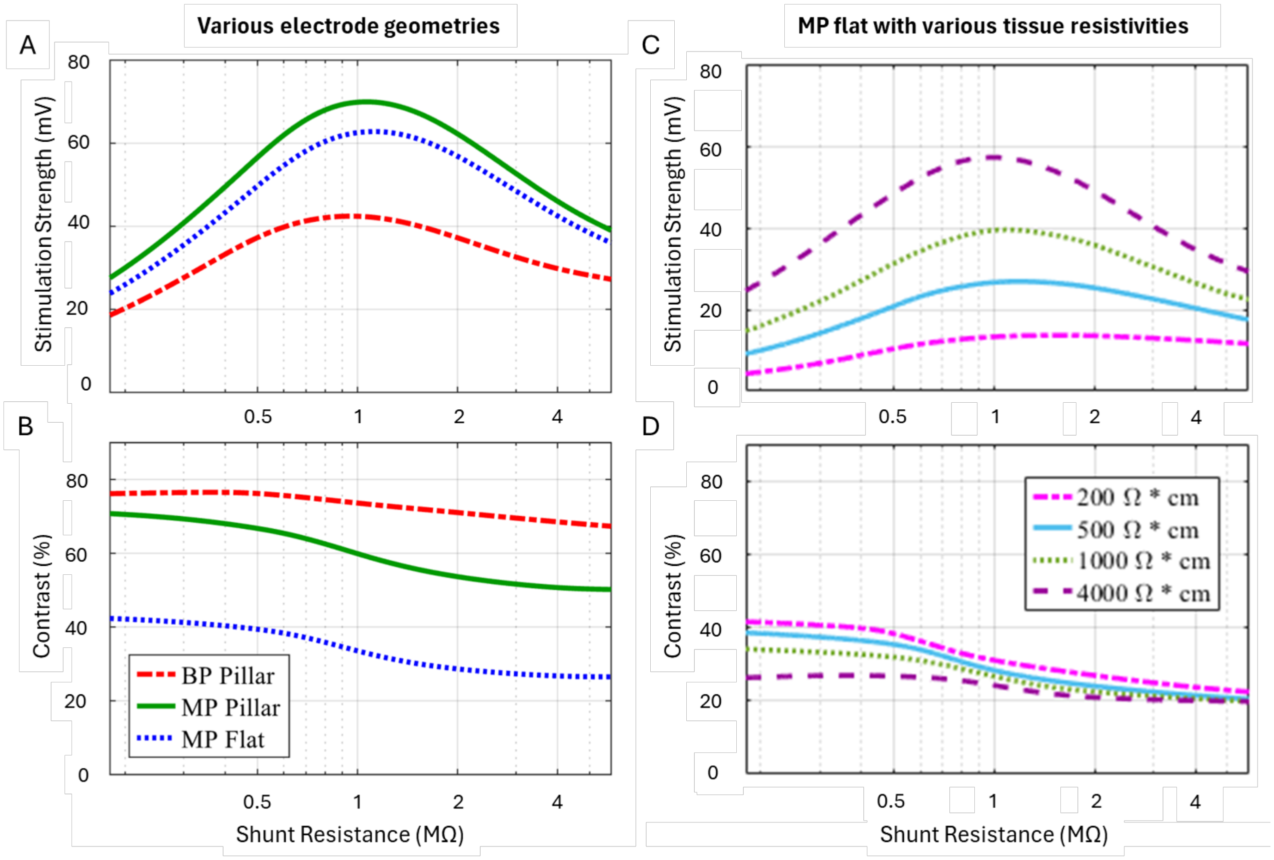

Figure S1. Modelled voltage drop and contrast across bipolar cells (35 - 87 μm above the implant) under Landolt C pattern with a gap width of 1.2 pixel size at 1.64 mW/mm^2 irradiance and repetition rate of 30 Hz, calculated for 40μm pixels array. (A) Potential drop across bipolar cells as a function of shunt resistance for various electrode configurations: monopolar flat, monopolar pillar and bipolar pillar. (B) Contrast between the gap and bright part of Landolt C. (C) Potential drop across bipolar cells as a function of shunt resistance for flat monopolar pixels, with various retinal resistivities. (D) Contrast between the gap and bright part of Landolt C for conditions shown in C.

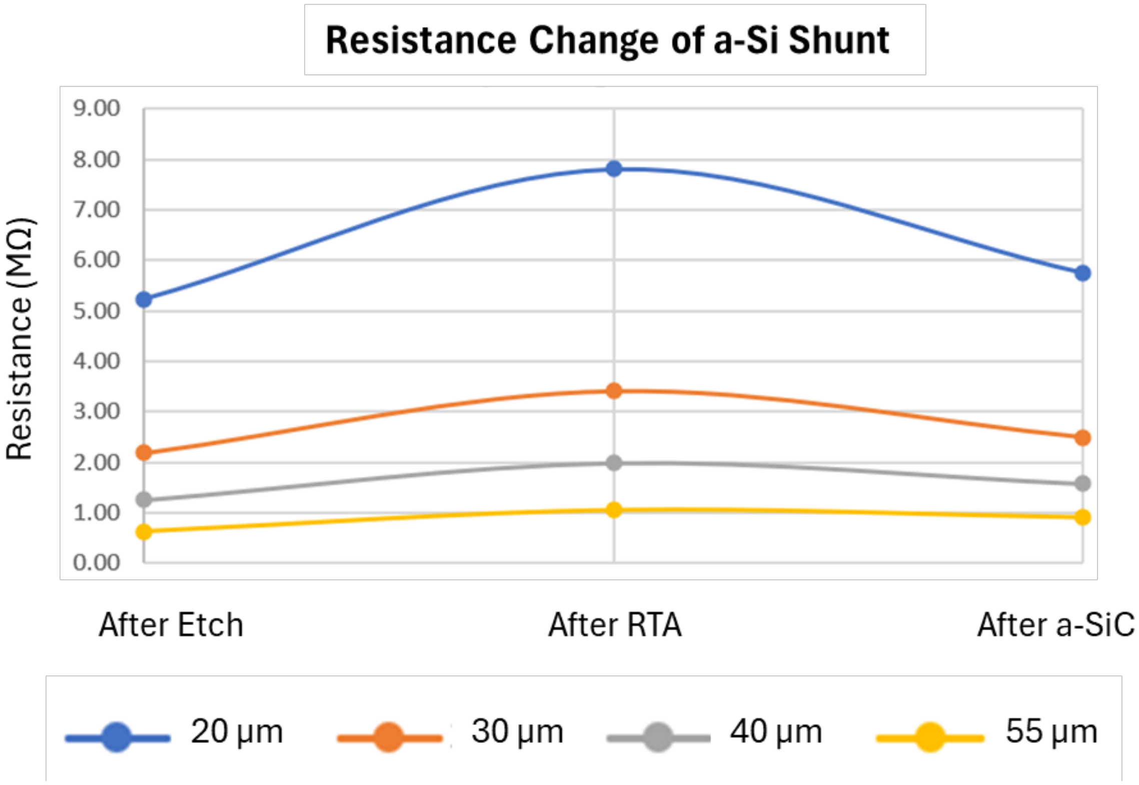

Figure S2. Resistance changes of the amorphous silicon layer corresponding to the shunt resistor patterns in the 20, 30, 40, and 55 µm pixels after Rapid Thermal Annealing (RTA) at 425 °C for 30 minutes to improve the ohmic contact between the titanium and amorphous silicon layer, and after adding the a-SiC anti-reflection layer for the insulation purpose.

Pixel size (μm)	No a-Si LPCVD, no shunt (μm^2)	a-Si LPCVD, no shunt (μm^2)	a-Si LPCVD, shunt (μm^2)
20	164	187	187
30	413	398	398
40	778	771	758
55	1545	1533	1506

Table S1. Photosensitive area of various pixels.

		20 μm Pixels			*30 μm Pixels*			*40 μm Pixels*		
**Model**	**Frequency, Hz**	**Bright (mV)**	**Dark (mV)**	**Contrast (%)**	**Bright (mV)**	**Dark (mV)**	**Contrast (%)**	**Bright (mV)**	**Dark (mV)**	**Contrast (%)**
No Shunt	2	**57.9 ± 0.2**	**44.2 ± 0.1**	**24**	**62.6 ± 0.2**	**48.3 ± 0.2**	**23**	**72.1 ± 0.2**	**56.5 ± 0.4**	22
No Shunt	30	**32.3 ± 0.2**	**25.2 ± 0.2**	**22**	**32.1 ± 0.1**	**26.1 ± 0.2**	**19**	**36.5 ± 0.2**	**29.2 ± 0.2**	20
Shunt	2	**48.6 ± 0.2**	**36.3 ± 0.2**	**25**	**50.5 ± 0.3**	**36.9 ± 0.2**	**27**	**60.5 ± 0.5**	**41.5 ± 0.2**	31
Shunt	30	**38.4 ± 0.2**	**28.7 ± 0.1**	**25**	**40.1 ± 0.4**	**28.6 ± 0.1**	**29**	**44.2 ± 0.2**	**32.0 ± 0.2**	28

Table S2. Peak electric potential at the beginning of the 10 ms pulse 20μm above the device in diluted saline (1.56 mS, 640 Ohm*cm), generated by various photodiode arrays under 1.64 mW/mm^2 illumination of Landolt C pattern with a gap width of 48μm, at pulse frequencies of 2 and 30 Hz.
